# Comparison of Body Mass Index and Fat Indices in Predicting the Severity of Nonalcoholic Fatty Liver Disease Among Children Who Are Overweight and Obese

**DOI:** 10.3389/fped.2021.724426

**Published:** 2021-08-27

**Authors:** Hsun-Chin Chao, Hsin-Yeh Lin

**Affiliations:** ^1^Division of Pediatric Gastroenterology, Department of Pediatrics, Chang Gung Children's Medical Center, Chang Gung Memorial Hospital, Taoyuan City, Taiwan; ^2^Chang Gung University College of Medicine, Taoyuan City, Taiwan

**Keywords:** abdominal fat, body mass index, children, fatty liver, waist circumference

## Abstract

**Background:** Information of the relationships between body mass parameters and the severity of fatty liver is deficient in pediatric nonalcoholic fatty liver disease (NAFLD).

**Methods:** The relationships between body mass parameters (waist circumference [WC], body mass index [BMI], and abdominal subcutaneous fat thickness [ASFT]) and the severity of fatty liver were prospectively evaluated in pediatric patients who are overweight or obese, suffering from NAFLD. Ultrasonography was performed to assess fatty liver and its severity on a three-grade scale (low-grade fatty liver [LGFL], grade 1 or 2; high-grade fatty liver [HGFL], grade 3).

**Results:** A total of 110 subjects (55 LGFL and 55 HGFL) aged 6.2–17.9 years were included. The WC, BMI, and ASFT values were significantly higher in the HGFL group compared to those in the LGFL group (*p* = 0.00004, 0.01, and 0.04, respectively). WC had the greatest power to predict HGFL under receiver-operating characteristic curve analyses and was positively correlated with the severity of fatty liver in subjects aged 6–12-year old and 13–17-year old (*p* = 0.007, and 0.0039, respectively). ASFT showed a positive correlation with the severity of fatty liver in subjects aged 13–17-year old (*p* = 0.04).

**Conclusions:** WC, BMI, and ASFT are predictive of severe NAFLD among children who are overweight and obese; particularly, WC has the most predictive accuracy. Among the parameters, WC and ASFT are predictive in specific age groups.

## Introduction

Nonalcoholic fatty liver disease (NAFLD) is a common cause of chronic liver disease worldwide, and its prevalence continues to increase with the growing epidemic of obesity (1). NAFLD can manifest as simple steatosis without the evidence of cell injury, which can progress to nonalcoholic steatohepatitis, cirrhosis (2) and eventually to hepatocellular carcinoma (3).

Obesity is a major public health and a medical care problem. The rates of obesity in children and adolescents are rapidly increasing, and the WHO has classified obesity as a disease (4). The amount of body fat itself is important; however, the distribution of body fat, particularly abdominal obesity, is a crucial risk factor for metabolic syndrome and cardiovascular disease (5). Furthermore, the prevalence of NAFLD in children is increasing; hence, determining their abdominal fat content is crucial for evaluating obesity. Visceral fat plays an essential role in the pathogenesis and progression of NAFLD (6, 7).

NAFLD is usually identified by abnormal liver tests, imaging studies, and liver biopsy (8). Ultrasound (US) of the liver is the most common technique for screening fatty liver in the general population (9); it is a fast, readily available, and relatively reliable method to diagnose fatty liver when Fibroscan is not available. It is recommended to investigate NAFLD in clinical settings in cases in which their changes are observed in patients through US. Several biochemical markers such as aspartate transaminase (AST), alanine transaminase (ALT), lipid profile, fasting blood sugar, and fasting insulin level are associated with NAFLD (10, 11). In clinical settings, the measurement of aminotransferases, blood lipids, and insulin resistance is often used to detect NAFLD and metabolic syndrome (11). The AST and ALT values are commonly used to assess the severity of NAFLD (12, 13).

There is a lack of investigations in the comparison of body mass index (BMI) and fat indices on the severity of fatty liver in pediatric NAFLD. The aim of this study was to investigate the demographic, body mass parameters, and liver function parameters and to compare various body mass parameters in predicting the severity of NAFLD among children who are overweight and obese. We further categorized the patients into two different age groups and tried to determine the most predictive body mass parameter.

## Materials and Methods

### Study Design

A 1-year prospective cross-sectional study on the correlations between body mass parameters (waist circumference [WC], BMI, abdominal subcutaneous fat thickness [ASFT]) and the severity of NAFLD was conducted in children aged between 6 and 18 years. Pediatric patients who are overweight or obese, who visited the Pediatric Gastroenterology Outpatient Department of Chang Gung Memorial Hospital, and who were diagnosed with NAFLD were enrolled. The severity of NAFLD was assessed by the grading of fatty liver on US. The correlations among WC, BMI, and ASFT values, the serum levels of AST and ALT, and the severity of fatty liver were evaluated.

### Definitions of Overweight and Obesity

Using sex-specific BMI-for-age curves, overweight and obesity were defined as one SD of the mean BMI and two SDs of age and sex according to the WHO growth reference for school-aged children and adolescents (14).

### Diagnosis of NAFLD and Exclusion Criteria

The diagnosis of NAFLD was based on the practice guidelines of the American Gastroenterological Association, the American Association for the Study of Liver Diseases, and the American College of Gastroenterology. These criteria are evidence of hepatic steatosis on US; no significant alcohol consumption, no other etiologies for hepatic steatosis, and no coexisting causes of chronic liver diseases (15) were observed. Patients who consumed alcohol and those with coexisting etiologies of chronic liver diseases (chronic viral hepatitis, hemochromatosis, infiltrative diseases, autoimmune liver diseases, metabolic diseases, Wilson's disease, hereditary diseases, and iatrogenic causes) were excluded. As glycogenic hepatopathy could occur in the patients with type 1 diabetes mellitus, causing elevation of liver enzymes, we excluded those patients with diabetes mellitus from the study to avoid the resulting bias.

### Categorization and Evaluation

The subjects were categorized into a low-grade fatty liver (LGFL) group (grade 1 or 2) and high-grade fatty liver (HGFL) group (grade 3). The subjects were divided into those aged 6–12 and 13–17 years to compare the differences of various body mass parameters in predicting HFGL between different age groups. We did not recruit the younger aged children (<6 years old) due to the BMI of a child reaching a natural nadir between 5 and 6 years of age (16). The inclusion might result in bias when evaluating the association between BMI and the severity of NAFLD and might increase the risk of measurement errors caused by their in-cooperation during US examination.

### US Assessment of Fatty Liver

US was performed using the Acuson Antares instrument (Siemens, Germany), equipped with a linear-array probe (6.15 MHz) and a convex-array probe (4.44 MHz). The US measurements were conducted by accredited pediatric gastroenterologists blinded to the laboratory data of subjects. The degree of fat accumulation in the liver was graded according to the echogenicity of the liver parenchyma, to the visibility of the vascular structure, and to the clarification of the diaphragm (17). In grade 1 fatty infiltration, a slightly increased hepatic echogenicity was noted with visible intrahepatic vessel borders and diaphragm. Grade 2 fatty infiltration was defined as a moderately increased hepatic echogenicity with a slightly impaired visualization of the intrahepatic vessels or diaphragm. Grade 3 was defined as a markedly increased hepatic echogenicity with the obscuration of intrahepatic vessel borders and diaphragm.

To minimize the diagnostic errors, we first examined the echogenicity of the hepatic parenchyma, intrahepatic vessel borders, and diaphragm. Then, liver echogenicity was compared with renal and pancreatic echogenicity to confirm hepatic hyperechogenicity. Finally, the details of the images were carefully reviewed to confirm the grading of fatty liver severity. Those participants lacking clear and detailed images were excluded from the study.

### Measurement of Body Mass Parameters and Liver Function

The height, WC, and weight of the subjects were measured to the nearest 0.1, 0.1, and 0.1 kg, respectively.

#### Waist Circumference

WC was measured halfway between the lower rib and the iliac crest when the subject was relaxed and exhaling normally (18). WC was measured as follows: a tape measure was placed immediately above the upper hip-bone parallel to the floor, was snug to the body but not tight as to compress the skin, and the subject exhaled and relaxed the abdomen during the measurement.

#### Body Mass Index

Weight and height were measured with the subject wearing light clothing and no shoes and were used to calculate BMI (weight [kg]/height [m^2^]). The BMI-for-age *z*-score was calculated using WHO Anthro software (WHO, Geneva, Switzerland) (14).

#### Abdominal Subcutaneous Fat Thickness

**Figure 1:**
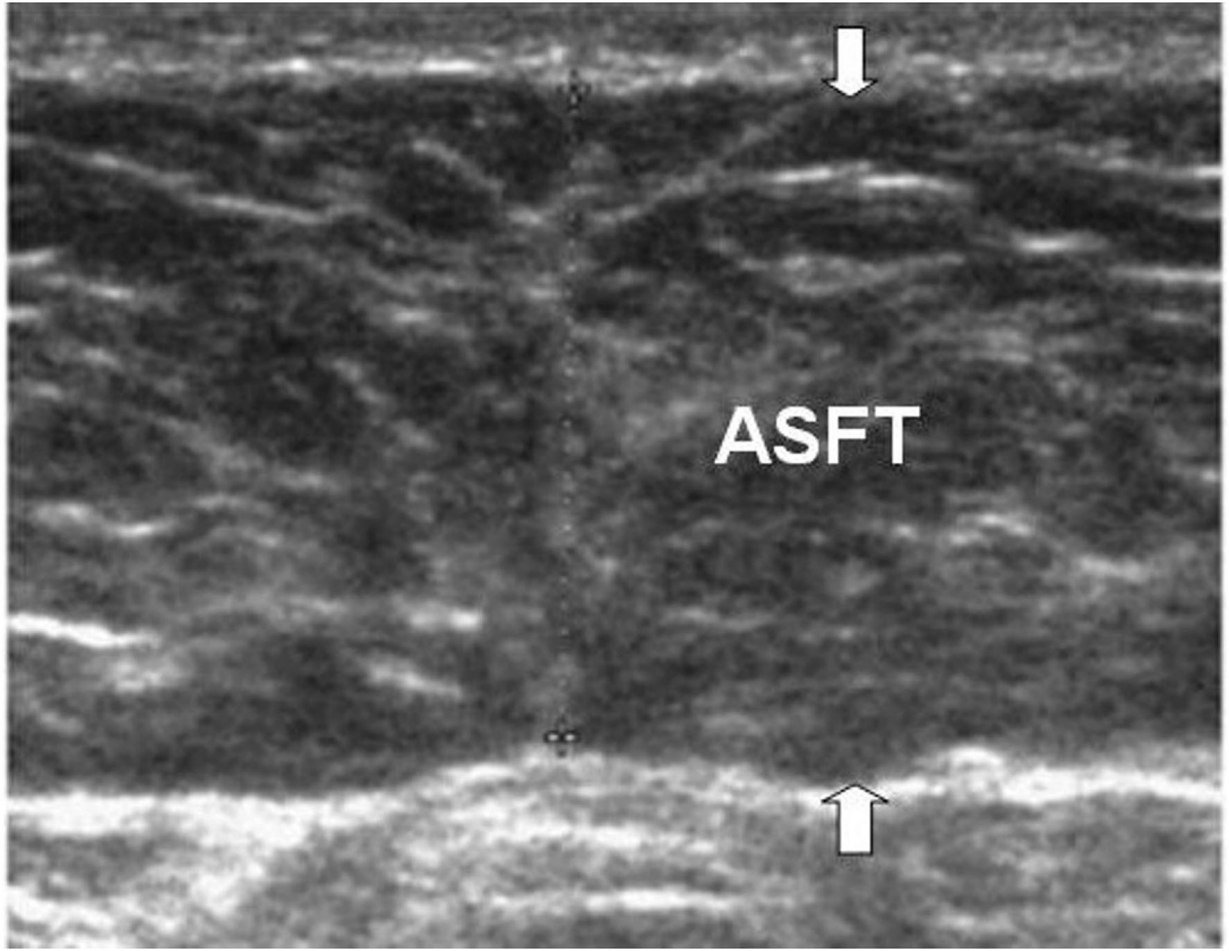
The figure illustrates the ASFT (arrows) with US. ASFT is measured using a linear probe, with the subject being perpendicular to the body surface in a supine position, and vertical scanning. The measurement of ASFT is only the thickness of the fat tissue instead of the traditional measurement as the distance from the skin to the linea alba.

The measurement of the ASFT was based on the US measurement procedure by Sogabe et al. (19). The measurement of ASFT was modified to measure only the thickness of the fat tissue instead of the measurement as the distance from the skin to the linea alba (Figure 1). ASFT was determined by US using a linear probe, with the subject being perpendicular to the body surface in a supine position, and vertical scanning was performed along the abdominal median from the xyphoid process to the umbilicus. To avoid fat compression errors, the US probe was placed above a given site with minimal pressure. The measurement procedure was performed by an experienced pediatric gastroenterologist (Dr. Hsun-Chin Chao), the measurements were repeated three times, and the mean of the three values was calculated.

#### Liver Chemistry

Venous blood samples were collected from each subject. To evaluate liver chemistry, AST and ALT levels were measured using enzymatic methods (ADVIA 1650, Siemens, Tarrytown, NY, USA).

### Statistical Analysis

Data entry and processing were conducted using Statistical Package for the Social Sciences (SPSS) v. 20 software (Armonk, NY, USA). Descriptive and inferential data analyses were performed using chi-squared tests and Student's *t*-tests. In addition, Student's *t*-test was used to compare the means of continuous variables between independent samples. Categorical data were analyzed using the chi-squared test. Linear regression analyses were conducted to evaluate the association between liver function and body mass parameters with the severity of fatty liver. Receiver-operating characteristic (ROC) curve analyses were conducted to assess the predictive power of body mass parameters for HGFL. An area under the curve (AUC) <0.5 suggests no discrimination; 0.7–0.8 is considered to be acceptable, 0.8–0.9 is considered to be excellent, and > 0.9 is considered to be outstanding (20). The level of statistical significance was set at *p* < 0.05. Continuous variables are expressed as means ± SDs, and categorical variables are expressed as percentages.

### Ethical Approval

This study protocol was in compliance with the Declaration of Helsinki and was approved by the ethics committee of Chang Gung Memorial Hospital (IRB No: 201800385B0). All the caregivers/guardian of the participants and those who were aged 13–17 years provided their written informed consent to participate in this study. Those participants who were aged 7–12 years signed the assent form.

## Results

### Patients and Subgroups

**Figure 2:**
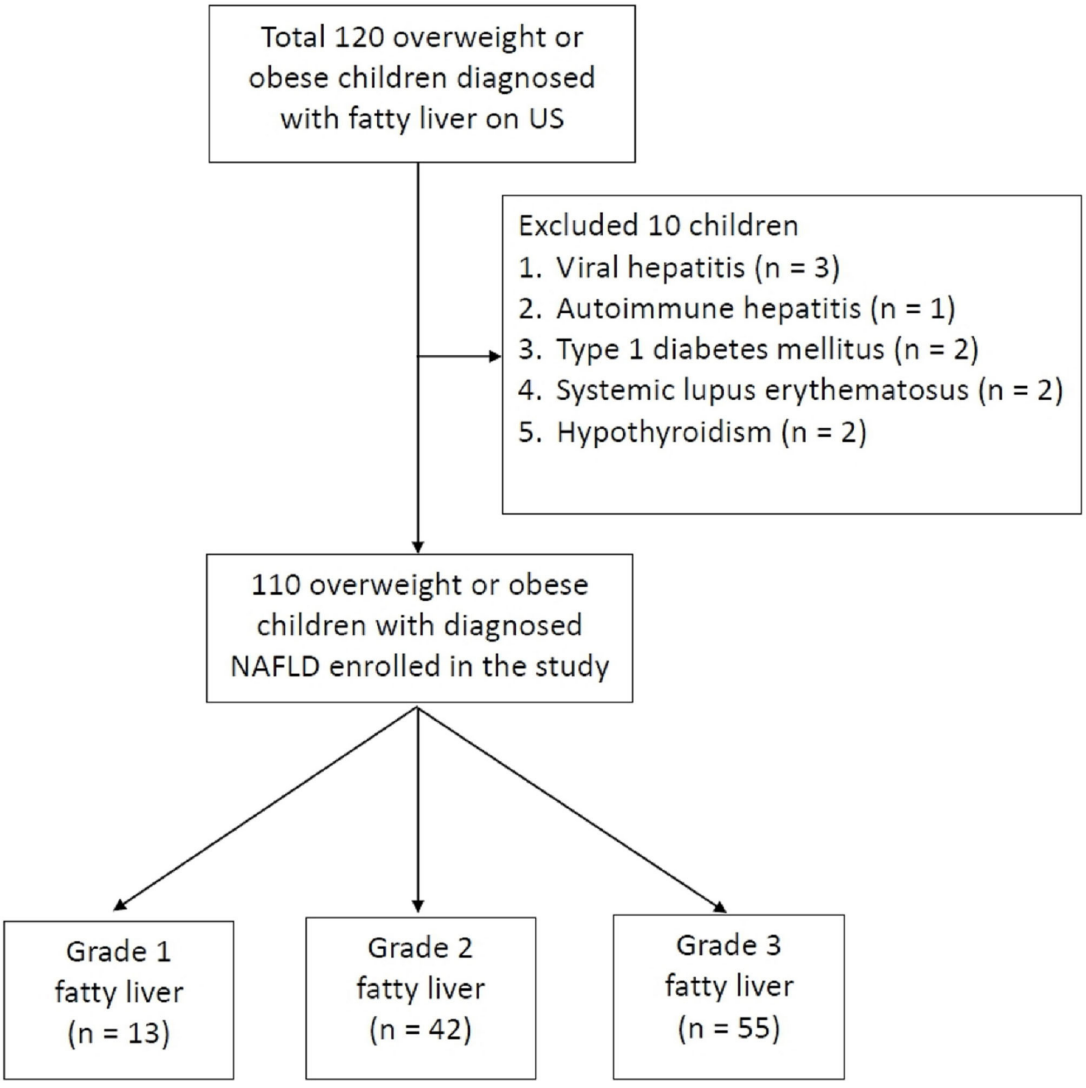
Flow chart shows patient inclusion, exclusion criteria, and classification. A total of 120 met the inclusion criteria; 10 were excluded for assorted medical conditions, with 110 patients finally being included in the study. The study patients are divided into three groups according to the grading of fatty liver on US.

Figure 2 displays the algorithm of patient inclusion and classification. In total, 120 pediatric patients who visited the Pediatric Gastroenterology Outpatient Department were identified to be overweight (or obesity) with fatty liver between May 2016 and April 2017. Of these 120 children, 10 were excluded. The excluded cases included viral hepatitis (*N* = *3*), autoimmune hepatitis (*N* = *1*), type 1 diabetes mellitus (*N* = *2*), systemic lupus erythematosus (*N* = *2*), and hypothyroidism (*N* = *2*). Finally, a total of 110 patients (82 men and 28 women) who were diagnosed with NAFLD were enrolled in the study. Their mean age was 12.9 ± 2.7 years (range 6.2–17.9 years). Among these 110 patients, 55 (50%) were categorized into the LGFL group [13 had grade 1 fatty liver (11.8%) and 42 had grade 2 (38.2%)] and 55 (50%) had grade 3 fatty liver who were categorized into the HGFL group.

### Demographics and Clinical Characteristics

Table 1 demonstrates the demographic data, overweight status, body mass parameters, and liver function of the patients. The mean WC and BMI of the patients were 94.21 ± 9.15 cm and 29.53 ± 3.88, respectively. A total of 23 patients (21%) were overweight and 87 (79%) were obese. The ASFT of the patients was 1.0–5.4 cm (mean, 3.38 ± 0.65 cm), and their mean AST and ALT levels were 47.84 ± 20.23 and 71.49 ± 41.01 U/L, respectively.

**Table 1:** Demographics and clinical characteristics of enrolled patients.

**Parameters**	**Values**
Number	110
Age (years, mean ± SD)	12.9 ± 2.7
Men (%)	82 (74.5%)
Overweight	23 (21 %)
Obese	87 (79%)
Body mass parameters	
WC (cm, mean ± SD)	94.21 ± 9.15
BMI (mean ± SD)	29.53 ± 3.88
BMI-for-age *z*-score (mean ± SD)	2.86 ± 1.00
ASFT (cm, mean ± SD)	3.38 ± 0.65
Liver function	
AST (U/L, mean ± SD)	47.84 ± 20.23
ALT (U/L, mean ± SD)	71.49 ± 41.01

*SD, standard deviation; WC, waist circumference; BMI, body mass index; ASFT, abdominal subcutaneous fat thickness*.

Table 2 shows the clinical characteristics of patients in the LGFL and HGFL groups. Men predominated in both the LGFL group (39 patients, 71%) and HGFL group (43 patients, 78%). There were no significant differences in age (*p* = 0.561), proportion of men (*p* = 0.511), or BMI-for-age *z*-score (*p* = 0.21) between the LGFL and HGFL groups. The AST, ALT, WC, BMI, and ASFT values were significantly higher in the HGFL group than in the LGFL group (*p* = 0.026, 0.0043, 0.00004, 0.01, and 0.04, respectively; Table 2).

**Table 2:** Differences of clinical characteristics between the LGFL and HGFL groups.

**Variables/ groups**	**LGFL**	**HGFL**	**Student's-*t* (95% CI)^a^/Chi-square^b^**	***P***
Number	55	55		
Mean of age (years)	12.8 ± 2.7	13.1 ± 2.7	−1.321,−0.7206^a^	0.561
Men (number, %)	39 (71%)	43 (78%)	0.431^b^	0.511
Body mass parameters				
WC (cm)	89.65 ± 7.21	98.77 ± 9.37	−13.35, −4.909^a^	0.00004^*^
BMI	28.33 ± 4.7	30.73 ± 5.2	−4.292, −0.521^a^	0.01^*^
BMI-for-age	2.67 ± 0.90	3.05 ± 1.08	−0.9947, −0.2242^a^	0.21
*z*-score				
ASFT	3.20 ± 0.63	3.54 ± 0.65	−0.6636, −0.01497^a^	0.04^*^
Liver function				
AST (U/L)	41.25 ± 16.69	54.42 ± 23.01	−24.74, −1.586^a^	0.026^*^
ALT (U/L)	56.84 ± 32.32	86.15 ± 46.87	−49.26, −9.355^a^	0.0043^*^

a Data of continuous variables were expressed as the mean ± SD and were analyzed by Student's t-test; the mean values were significantly different between variables (p <0.05^*^).

b Descriptive data were analyzed by Chi-squared test; the number (percentage) was significantly different between variables (p <0.05^*^).

*WC, waist circumference; LGFL, low-grade fatty liver; HGFL, high-grade fatty liver; BMI, body mass index; ASFT, abdominal subcutaneous fat thickness; CI, confidence interval*.

**Figure 3:**
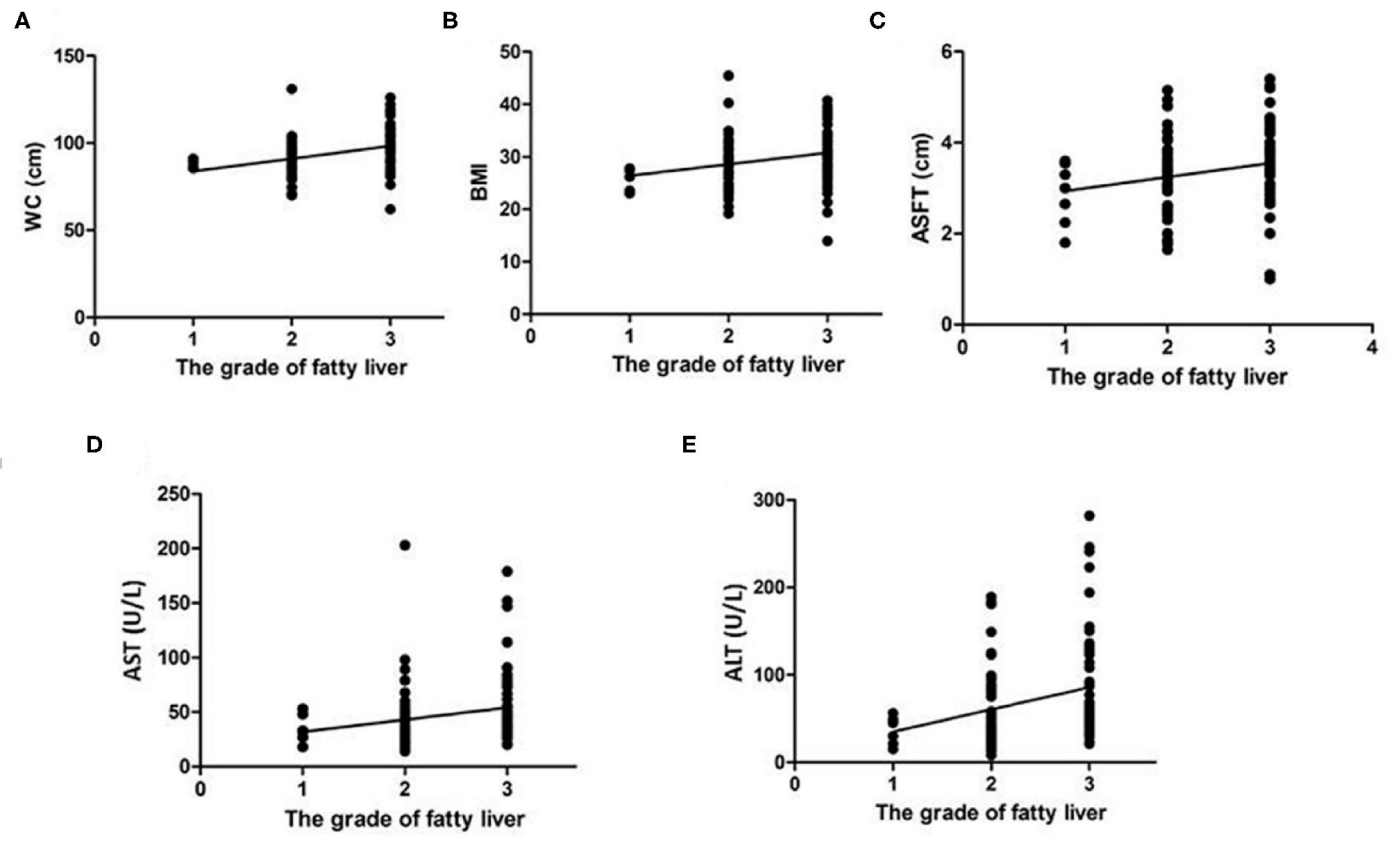
Linear regression analysis of the correlation of WC **(A)**, BMI **(B)**, ASFT **(C)**, AST **(D)**, and ALT **(E)** with the severity of fatty liver in children with NAFLD. The values of all the determinants are associated with the severity of fatty liver with statistical significance (*p* < 0.05).

Figure 3 shows the linear regression analysis of the relationship between WC, BMI, ASFT, AST, and ALT values and the severity of fatty liver; significant correlation of values with the severity of fatty liver was found (*p* < 0.0001, 0.0054, 0.0213, 0.0227, and 0.025, respectively).

### Demographics and Clinical Characteristics Among Different Age Groups

There were 52 patients in the 6–12-year group and 58 patients in the 13–17-year-old group. The mean ages of patients in the 6- to 12-year LGFL group, in the 13–17-year LGFL group, in the 6–12-year HGFL group, and in the 13–17-year HGFL group were 10.2 ± 1.4, 16 ± 1.3, 9.8 ± 1.6, and 15.4 ± 1.6 years, respectively. The men-to-women ratio was 4.2 (men, 81%) in the 6–12-year-old group and 2.2 (men, 69%) in the 13–17-year-old group. The numbers of patients in the 6–12-year LGFL group, in the 13–17-year LGFL group, in the 6–12-year HGFL group, and in the 13–17-year HGFL group were 29, 26, 23, and 32, respectively.

There were no significant differences in the mean age of both 6–12 and 13–17 year groups between the LGFL and HGFL groups (6–12 year groups: 95% CI: −0.3448–1.145, *p* = 0.286; 13–17 year groups: 95% CI: −0.2647–1.465, *p* = 0.170).

Table 3 demonstrates the values of body mass parameters between LGFL and HGFL in different age groups. The WC was significantly higher in subjects with HGFL in both age groups (*p* = 0.007 and 0.0039, respectively) (Table 3). The ASFT was significantly correlated with the severity of NAFLD in the 13–17-year-old group (*p* = 0.04) but not in the 6–12-year group (*p* = 0.67) (Table 3).

**Table 3:** Values of body mass parameters between LGFL and HGFL in different age groups.

**Variables/groups**	**LGFL**	**HGFL**	**95% CI**	***P***
BMI				
6–12 yrs	27.52 ± 4.31	29.81 ± 5.19	−4.937, 0.3613	0.089
13–17 yrs	29.23 ± 5.06	31.40 ± 5.25	−4.905, 0.5632	0.117
WC (cm)				
6–12 yrs	87.38 ± 5.88	94.57 ± 7.41	−12.34, −2.044	0.007^a^
13–17 yrs	92.18 ± 8.54	101.80± 10.35	−16.03, −3.214	0.0039^*^
ASFT				
6–12 yrs	3.22 ± 0.61	3.30 ± 0.49	−0.5102, 0.334	0.67
13–17 yrs	3.21 ± 0.64	3.72 ± 0.73	−0.998, −0.0238	0.04^*^

Data of continuous variables were expressed as the mean ± SD and were analyzed by Student's t-test; the mean values were significantly different between variables (p < 0.05^*^).

*WC, circumference; LGFL, low-grade fatty liver; HGFL, high-grade fatty liver, CI, confidence interval; BMI, body mass index; ASFT, abdominal subcutaneous fat thickness*.

### Predictability of Body Mass Parameters for HGFL

**Figure 4:**
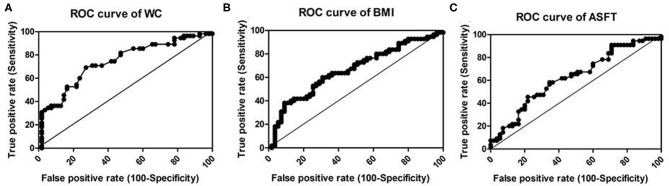
Receiver–operator characteristic curve (ROC) to illustrate the values of WC **(A)**, BMI **(B)**, and ASFT **(C)** as predictors of HGFL in children with NAFLD. The line in the diagonal presents the ROC curve of a random predictor (AUC: 0.5). Among the three body mass parameters, WC has the greatest power to predict HGFL.

Figure 4 displays the ROC curve analysis of the predictive values of WC (A), BMI (B), and ASFT (C) in HGFL in children with NAFLD. Among the three body mass parameters, WC (AUC, 0.741; *P* < 0.001) had a greater power to predict HGFL than BMI (AUC, 0.662; *p* = 0.003) or ASFT (AUC, 0.626; *p* = 0.022) (Table 4).

**Table 4:** ROC curve analysis of body mass parameters (WC, BMI, and ASFT) to identify HGFL.

**Body mass parameters**	**Detection of HGFL**	**AUC**	**Std. error**	**95% CI**		***P***
				**Lower bound**	**Upper bound**	
WC	94.5	0.741	0.047	0.648	0.834	<0.001^*^
ASFT	3.4575	0.626	0.053	0.522	0.731	0.022^*^
BMI	29.5744	0.662	0.052	0.560	0.764	0.003^*^

* *Significance of difference between variables (p < 0.05)*.

*ROC, receiver-operating characteristic; WC, waist circumference; BMI, body mass index; ASFT, abdominal subcutaneous fat thickness; HGFL, high-grade fatty liver; AUC, area under the curve; Std, standard*.

## Discussion

The present study is the first to compare the predictability of BMI and fat indices for HGFL in children who are overweight and obese. In this study, we found that WC, BMI, and ASFT values were correlated with the severity of fatty liver in children with NAFLD. Among these body mass parameters, WC was the only predictor for severe fatty liver in different age groups (6–12 and 13–17 years), while ASFT was predictive of severe fatty liver in those 13–17 years of age.

Several methods using noninvasive detection of NAFLD by anthropometry and circulating biomarkers have been established to predict the presence of NAFLD in adults and children (21–23). WC and BMI are usually measured to detect the metabolic syndrome, the relationships of NAFLD with BMI, and WC have been reported in several publications (24, 25). Compared to reports in adults, there are relatively limited publications regarding the relationship between NAFLD and anthropometric measurements in children. BMI was recommended as a predictor of hepatic fibrosis in children with nonalcoholic steatohepatitis (26, 27). A positive correlation between WC and NAFLD has been reported in childhood (24, 28). NAFLD is strongly linked to obesity, as its prevalence is 80% in patients with obesity and 16% in individuals with a normal BMI and without metabolic risk factors (29). Abdominal obesity is a vital determinant in NAFLD pathogenesis due to its association with insulin resistance and a possible source of free fatty acids (30). Although BMI is the standard for determining obesity, it does not reflect the distribution of body fat. Indeed, BMI is insufficient for assessing abdominal obesity in children because the amount of body fat varies with growth and development (31, 32). In this study, BMI, but not BMI-for-age *z*-score, was positively correlated with the severity of fatty liver. In addition, we found that BMI was unable to predict the HGFL among different age groups. WC is more strongly correlated with metabolic syndrome components than the percentage body fat or BMI (33), and the risk of NAFLD increases with increasing WC (34, 35). Therefore, WC could facilitate the identification of children who need a physician supervision because of the risk of acquiring metabolic disorders. In this study, only WC was positively correlated with the severity of fatty liver in both younger (6–12 years) and older age (13–17 years) groups. Furthermore, we found that abdominal fat provided information regarding the risk of severe fatty liver in children with NAFLD.

CT, MRI, and dual-energy X-ray absorptiometry (DEXA) are frequently used to measure the abdominal obesity in adults (36–39). However, their use in routine clinical practice is hampered by the associated cost, time, radiation exposure, and diverse contraindications. Abdominal US is a noninvasive, easy, and safe tool to assess hepatic diseases; it is widely used for screening asymptomatic patients with incidental elevation of liver enzymes or patients with suspected NAFLD. The US findings of suspected NAFLD include hepatomegaly, diffuse increases in the echogenicity of the liver parenchyma, and vascular blunting. With the development of computer technology, nowadays, it is possible to quantitatively determine the liver fat content by the US hepatic/renal ratio (40, 41). In addition, US provides a reliable and convenient means of quantifying the visceral fat thickness (VFT) and ASFT. There is a highly significant correlation between the amount of intra-abdominal adipose tissue determined by CT and US (42, 43). Visceral adipose tissue is associated with the degree of hepatic fat infiltration, even in nonobese adults (44). In a study of adolescent patients with NAFLD, visceral adiposity was associated with the severity of hepatic steatosis in patients who are mostly men (45). VFT, rather than the body weight or body fat percentage, is reportedly an independent risk factor for NAFLD (46), and an increased VFT is a prognostic factor for insulin resistance and NAFLD in children (47). The abdominal-wall fat index, defined as the ratio of the preperitoneal fat thickness ÷ the subcutaneous fat thickness measured using US, is related to NAFLD and/or advanced hepatic fibrosis in patients with diabetes (48).

Compared to the reports on adult patients, there are evidently limited publications about the relationship between NAFLD and anthropometric measurements of abdominal fat in childhood. Visceral and subcutaneous fat measurements provide information regarding metabolic dysregulation in children affected by obesity and NAFLD (49). The visceral fat measured using MRI or DEXA found that visceral fat was an independent factor of NAFLD or hepatic fibrosis in children and adolescents with obesity (50, 51). In our study, we used the US measurement of ASFT instead of VFT because the measurement of VFT using US can be challenging in patients with obesity due to attenuated US images in the deep portion, whereas the measurement of ASFT is easier and has less error because the measurement site is closer to the body surface. Besides, the measurements of ASFT by measuring the fat tissue only could accurately represent the true thickness of ASFT. In this study, we found that ASFT measured using US was positively correlated with the severity of fatty liver in overweight children with NAFLD, particularly in the 13–17 years of age.

Proton magnetic resonance spectroscopy (1H-MRS) is considered the most accurate technique of detection and quantification of hepatic steatosis (52). US elastography is a simple and efficient method, which is being increasingly used to diagnose pathological variations in liver stiffness and steatosis [controlled attenuation parameter (CAP)]. However, the interpretation of results can differ from patient to patient, depending on the liver disease etiology and the underlying comorbidities that should be considered (53).

The strengths of this study are the random recruitment of the children who are overweight or obese, as well as the standardized, single-center measurements. This study provides age- and sex-related reference charts for widely used body mass parameters for NAFLD in children and adolescents aged 6–17 years who are overweight and obese. The simple method of measuring ASFT using US enables clinicians an easy and fast evaluation of the references of their patients for assessing the severity of NAFLD in the clinical setting.

This study had several limitations. First, the cross-sectional design of the study prevented the assessment of age-related changes; thus, longitudinal analyses are needed. Second, our analyses were performed in Taiwan, and the ASFT values may differ among ethnicities. Third, we did not assess the correlations between WC, BMI, and ASFT and pathologically proven steatohepatitis or did not perform comparisons with MRI, DXEA, or 1H-MRS findings.

Growing evidence indicates that the US elastography technique is a reliably noninvasive method to assess the liver fibrosis stage and the steatosis grade in adult patients with NAFLD. Yet, there are still controversial results when applying to pediatric patients. We are currently accumulating our cases; future research is expected to compare the current results with the US elastography.

## Conclusion

WC, BMI, and ASFT are associated with the severity of fatty liver in NAFLD among children who are overweight and obese. HGFL is best predicted by WC, which is the only predictive parameter for severe fatty liver in different age groups (6–12 and 13–17 years). In addition, ASFT measured by US could be used to predict severe fatty liver, particularly in older children (13–17 years).

## Data Availability Statement

The original contributions presented in the study are included in the article/supplementary materials, further inquiries can be directed to the corresponding author.

## Ethics Statement

The studies involving human participants were reviewed and approved by the ethics committee of Chang Gung Memorial Hospital (IRB No: 201800385B0). Written informed consent to participate in this study was provided by the participants' legal guardian/next of kin.

## Author Contributions

H-CC contributed to the conception and design of the work and performed the statistical analysis. H-YL conducted the analysis and interpretation of the data. H-CC and H-YL wrote the draft of the manuscript. H-CC had full access to all the data in this study and had final responsibility for the decision to submit for publication. All authors contributed to manuscript revision, read, and approved the submitted version.

## Conflict of Interest

The authors declare that the research was conducted in the absence of any commercial or financial relationships that could be construed as a potential conflict of interest.

## Publisher's Note

All claims expressed in this article are solely those of the authors and do not necessarily represent those of their affiliated organizations, or those of the publisher, the editors and the reviewers. Any product that may be evaluated in this article, or claim that may be made by its manufacturer, is not guaranteed or endorsed by the publisher.

## References

[1] Vernon G Baranova A Younossi Z. Systematic review: the epidemiology and natural history of non-alcoholic fatty liver disease and non-alcoholic steatohepatitis in adults. Aliment Pharmacol Ther. (2011) 34:274–85. 10.1111/j.1365-2036.2011.04724.x 21623852

[2] Farrell G Larter C. Nonalcoholic fatty liver disease: from steatosis to cirrhosis. Hepatology. (2006) 43:S99–112. 10.1002/hep.20973 16447287

[3] White D Kanwal F El-Serag H. Association between nonalcoholic fatty liver disease and risk for hepatocellular cancer, based on systematic review. Clin Gastroenterol Hepatol. (2012) 10:1342–59. 10.1016/j.cgh.2012.10.001 23041539 PMC3501546

[4] Obesity: preventing and managing the global epidemic. Report of a WHO consultation. World Health Organ Tech Rep Ser. (2000) 894:1–253. 11234459

[5] Hojgaard B Olsen KR Sogaard J Gyrd-Hansen D Sorensen TI. Obesity related health care costs assessed from BMI or waist circumference: secondary publication. Ugeskr Laeger. (2009) 171:3068–71. 19866505

[6] van der Poorten D Milner KL Hui J Hodge A Trenell MI Kench JG. Visceral fat: a key mediator of steatohepatitis in metabolic liver disease. Hepatology. (2008) 48:449–57. 10.1002/hep.22350 18627003

[7] Petta S Amato M Cabibi D Cammà C Pizzolanti G Barcellona MR. Visceral adiposity index is associated with significant fibrosis in patients with non-alcoholic fatty liver disease. Aliment Pharmacol Ther. (2012) 35:238–47. 10.1111/j.1365-2036.2011.04929.x 22117531

[8] Ahmed M. Non-alcoholic fatty liver disease in 2015. World J Hepatol. (2015) 7:1450–9. 10.4254/wjh.v7.i11.1450 26085906 PMC4462685

[9] Bellentani S Scaglioni F Marino M Bedogni G. Epidemiology of non-alcoholic fatty liver disease. Dig Dis. (2010) 28:155–61. 10.1159/000282080 20460905

[10] Anderson EL Howe LD Jones HE Higgins JP Lawlor DA Fraser A. The prevalence of non-alcoholic fatty liver disease in children and adolescents: A systematic review and meta-analysis. PLoS ONE. (2015) 10:e0140908. 10.1371/journal.pone.0140908 26512983 PMC4626023

[11] Alterio A Alisi A Liccardo D Nobili V. Non-alcoholic fatty liver and metabolic syndrome in children: A vicious circle. Horm Res Paediatr. (2014) 82:283–9. 10.1159/000365192 25324136

[12] McLernon DJ Donnan PT Sullivan FM Roderick P Rosenberg WM Ryder SD. Prediction of liver disease in patients whose liver function tests have been checked in primary care: Model development and validation using population-based observational cohorts. BMJ Open. (2014) 4:e004837. 10.1136/bmjopen-2014-004837 24889852 PMC4054629

[13] Swain M Nath P Parida PK Narayan J Padhi PK Pati GK. Biochemical profile of nonalcoholic fatty liver disease patients in eastern India with histopathological correlation. Indian J Clin Biochem. (2017) 32:306–14. 10.1007/s12291-016-0612-7 28811690 PMC5539007

[14] The WHO reference for children aged 5–19 years is: de Onis M Onyango AW Borghi E Siyam A Nishida C Siekmann J. Development of a WHO growth reference for school-aged children and adolescents. Bulletin of the World Health Organization. (2007) 85:660–7. 10.2471/BLT.07.043497 18026621 PMC2636412

[15] Skelton J Cohen G. Obesity in Wyllie R, Hyams JS, Kay M. Pediatric Gastrointestinal and Liver Disease. 5th edn. Elsevier Inc: Philadelphia. (2015) p. 156

[16] Chalasani N Younossi Z Lavine JE. Diehl AM Brunt EM Cusi K. The diagnosis and management of non-alcoholic fatty liver disease: practice guideline by the American Gastroenterological Association, American Association for the Study of Liver Diseases, and American College of Gastroenterology. Gastroenterology. (2012) 142:1592–609. 10.1053/j.gastro.2012.04.001 22656328

[17] Saadeh S Yunossi Z Remer E Gramlich T Ong JP Hurley M. The utility of radiological imaging in nonalcoholic fatty liver disease. Gastroenterology. (2002) 123:745–50. 10.1053/gast.2002.35354 12198701

[18] Glasser N Zellner K Kromeyer-Hauschild K Validity of body mass index and waist circumference to detect excess fat mass in children aged 7-14 years. Eur J Clin Nutr. (2011) 65:151–9. 10.1038/ejcn.2010.245 21048772

[19] Sogabe M Okahisa T Hibino S Yamanoi A. Usefulness of differentiating metabolic syndrome into visceral fat type and subcutaneous fat type using ultrasonography in Japanese males. J Gastroenterol. (2012) 47:293–9. 10.1007/s00535-011-0489-4 22065161

[20] Shewhart WA Wilks SS. Assessing the Fit of the Model. In: Hosmer DW Lemeshow S, editors. Applied Logistic Regression2nd edn. New York: John Wiley and Sons. (2000), p.143–202.

[21] Maffeis C Banzato C Rigotti F Nobili V Valandro S Manfredi R. Biochemical parameters and anthropometry predict NAFLD in obese children. J Pediatr Gastroenterol Nutr. (2011) 53:590–3. 10.1097/MPG.0b013e31822960be 21697744

[22] Zhang HX Xu XQ Fu JF Lai C Chen XF. Predicting hepatic steatosis and liver fat content in obese children based on biochemical parameters and anthropometry. Pediatr Obes. (2015) 10:112–7. 10.1111/ijpo.232 24903159

[23] Eng K Lopez R Liccardo D Nobili V Alkhouri N A. non-invasive prediction model for non-alcoholic steatohepatitis in paediatric patients with non-alcoholic fatty liver disease. Dig Liver Dis. (2014) 46:1008–13. 10.1016/j.dld.2014.07.016 25106814

[24] Zelber-Sagi S Nitzan-Kaluski D Halpern Z Oren R. Prevalence of primary non-alcoholic fatty liver disease in a population based study and its association with biochemical and anthropometric measures. Liver Int. (2006) 26: 856–63. 10.1111/j.1478-3231.2006.01311.x 16911469

[25] Motamed N Sohrabi M Ajdarkosh H Hemmasi G Maadi M Sayeedian FS. Fatty liver index vs. waist circumference for predicting non-alcoholic fatty liver disease. World J Gastroenterol. (2016) 22:3023–30. 10.3748/wjg.v22.i10.3023 26973398 PMC4779925

[26] Iacobellis A Marcellini M Andriulli A Perri F Leandro G Devito R. Non-invasive evaluation of liver fibrosis in paediatric patients with nonalcoholic steatohepatitis. World J Gastroenterol. (2006) 12:7821–5. 10.3748/wjg.v12.i48.7821 17203527 PMC4087549

[27] Liang S Cheng X Hu Y Song R Li G. Insulin-like growth factor 1 and metabolic parameters are associated with nonalcoholic fatty liver disease in obese children and adolescents. Acta Paediatr. (2017) 106:298–303. 10.1111/apa.13685 27889912

[28] Clemente AP Netto BD de Carvalho-Ferreira JP da Silveira Campos RM de Piano Ganen A Tock L. Waist circumference as a marker for screening nonalcoholic fatty liver disease in obese adolescents. Rev Paul Pediatr. (2016) 34:47–55. 10.1016/j.rppede.2015.10.004 26830602 PMC4795721

[29] Prentice AM Jebb SA. Beyond body mass index. Obes Rev. (2001) 2:141–7. 10.1046/j.1467-789x.2001.00031.x 12120099

[30] Kim D Touros A Kim WR. Nonalcoholic Fatty Liver Disease and Metabolic Syndrome. Clin Liver Dis. (2018) 22:133–40. 10.1016/j.cld.2017.08.010 29128053

[31] Milić S Lulić D Štimac D. Non-alcoholic fatty liver disease and obesity: biochemical, metabolic and clinical presentations. World J Gastroenterol. (2014) 20:9330–7. 10.3748/wjg.v20.i28.9330 25071327 PMC4110564

[32] Ju DY Choe YG Cho YK Shin DS Yoo SH Yim SH. The influence of waist circumference on insulin resistance and nonalcoholic fatty liver disease in apparently healthy Korean adults. Clin Mol Hepatol. (2013) 19:140–7. 10.3350/cmh.2013.19.2.140 23837138 PMC3701846

[33] Shen W Punyanitya M Chen J. Gallagher D Albu J Pi-Sunyer X. Waist circumference correlates with metabolic syndrome indicators better than percentage fat. Obesity (Silver Spring). (2006) 14:727–36. 10.1038/oby.2006.83 16741276 PMC1894647

[34] Janiszewski PM Janssen I Ross R. Does waist circumference predict diabetes and cardiovascular disease beyond commonly evaluated cardiometabolic risk factors? Diabetes Care. (2007) 30:3105–9. 10.2337/dc07-0945 17712026

[35] Rocha R Cotrim HP Carvalho FM Siqueira AC Braga H Freitas LA. Body mass index and waist circumference in non-alcoholic fatty liver disease. J Hum Nutr Diet. (2005) 18:365–70. 10.1111/j.1365-277X.2005.00634.x 16150132

[36] Yoshizumi T Nakamura T Yamane M Islam AH Menju M Yamasaki K. Abdominal fat: standardized technique for measurement at CT. Radiology. (1999) 211:283–6. 10.1148/radiology.211.1.r99ap15283 10189485

[37] Kodama Y Ng CS Wu TT Curley SA Abdalla EK Vauthey JN. Comparison of CT methods for determining the fat content of the liver. AJR Am J Roentgenol. (2007) 188:1307–12. 10.2214/AJR.06.0992 17449775

[38] Schwenzer NF Springer F Schraml C Stefan N Machann J Schick F. Non-invasive assessment and quantification of liver steatosis by ultrasound, computed tomography and magnetic resonance. J Hepatol. (2009) 51:433–45. 10.1016/j.jhep.2009.05.023 19604596

[39] Micklesfield LK Goedecke JH Punyanitya M Wilson KE Kelly TL. Dual-energy X-ray performs as well as clinical computed tomography for the measurement of visceral fat. Obesity (Silver Spring). (2012) 20:1109–14. 10.1038/oby.2011.367 22240726 PMC3343346

[40] Zhang B Ding F Chen T Xia LH Qian J Lv GY. Ultrasound hepatic/renal ratio and hepatic attenuation rate for quantifying liver fat content. World J Gastroenterol. (2014) 20:17985–92. 10.3748/wjg.v20.i47.17985 25548498 PMC4273150

[41] Xia MF Yan HM He WY Li XM Li CL Yao XZ. Standardized ultrasound hepatic/renal ratio and hepatic attenuation rate to quantify liver fat content: an improvement method. Obesity (Silver Spring). (2012) 20:444–52. 10.1038/oby.2011.302 22016092 PMC3270296

[42] Suzuki R Watanabe S Hirai Y Akiyama K Nishide T Matsushima Y. Abdominal wall fat index, estimated by ultrasonography, for assessment of the ratio of visceral fat to subcutaneous fat in the abdomen. Am J Med. (1993) 95:309–14. 10.1016/0002-9343(93)90284-V 8368228

[43] Kawamoto R Ohtsuka N Nakamura S Ninomiya D Inoue A. Preperitoneal fat thickness by ultrasonography and obesity-related disorders. J Med Ultrason. (2007) 34:93–9. 10.1007/s10396-007-0137-z 27278292

[44] Yu J Lv Y Di W Liu J Kong X Sheng Y. MiR-27b-3p Regulation in Browning of Human Visceral Adipose Related to Central Obesity. Obesity (Silver Spring). (2018) 26:387–96. 10.1002/oby.22104 29280351

[45] Ayonrinde OT Olynyk JK Beilin LJ Mori TA Pennell CE de Klerk N. Gender-specific differences in adipose distribution and adipocytokines influence adolescent nonalcoholic fatty liver disease. Hepatology. (2011) 53:800–9. 10.1002/hep.24097 21374659

[46] Andrade LJ Melo PR Paraná R Daltro C. Grading scale of visceral adipose tissue thickness and their relation to the nonalcoholic fatty liver disease. Arq Gastroenterol. (2014) 51:118–22. 10.1590/S0004-28032014000200009 25003263

[47] Jung JH Jung MK Kim KE Kwon AR Chae HW Yoon CS. Ultrasound measurement of pediatric visceral fat thickness: correlations with metabolic and liver profiles. Ann Pediatr Endocrinol Metab. (2016) 21:75–80. 10.6065/apem.2016.21.2.75 27462583 PMC4960018

[48] Fukuda K Seki Y Ichihi M Okada T Hirata A Kogita S. Usefulness of ultrasonographic estimation of preperitoneal and subcutaneous fat thickness in the diagnosis of nonalcoholic fatty liver disease in diabetic patients. J Med Ultrason. (2015) 42:357–63. 10.1007/s10396-015-0615-7 26576787

[49] Mager DR. YapJ, Rodriguez-Dimitrescu C, Mazurak V, Ball G, Gilmour S. Anthropometric measures of visceral and subcutaneous fat are important in thedetermination of metabolic dysregulation in boys and girls at risk for non-alcoholic fatty liver disease. Nutr Clin Pract. (2013) 28:101–11. 10.1177/0884533612454884 23042833

[50] Bille DS Chabanova E Gamborg M Fonvig CE Nielsen TR Thisted E. Liver fat content investigated by magnetic resonance spectroscopy in obese children and youths included in multidisciplinary treatment. Clin Obes. (2012) 2:41–9. 10.1111/j.1758-8111.2012.00038.x 25586046

[51] Silveira LS Monteiro PA Antunes Bde M Seraphim PM Fernandes RA Christofaro DG. Intra-abdominalfat is related to metabolic syndrome and non-alcoholic fat liver. Disease in obese youth. BMC Pediatr. (2013) 13:115. 23919592 10.1186/1471-2431-13-115 PMC3751250

[52] Bohte AE van Werven JR Bipat S Stoker J. The diagnostic accuracy of US, CT, MRI and 1H-MRS for the evaluation of hepatic steatosis compared with liver biopsy: a meta-analysis. Eur Radiol. (2011) 21:87–97. 10.1007/s00330-010-1905-5 20680289 PMC2995875

[53] Pu K Wang Y Bai S Wei H Zhou Y Fan J. Diagnostic accuracy of controlled attenuation parameter (CAP) as a non-invasive test for steatosis in suspected non-alcoholic fatty liver disease: a systematic review and meta-analysis. BMC Gastroenterol. (2019) 19:51. 10.1186/s12876-019-0961-9 30961539 PMC6454693

